# Ultra-specific discrimination of single-nucleotide mutations using sequestration-assisted molecular beacons†
†Electronic supplementary information (ESI) available: Details in experimental section and supporting tables and figures. See DOI: 10.1039/c6sc03048c



**DOI:** 10.1039/c6sc03048c

**Published:** 2016-09-19

**Authors:** Shichao Hu, Wei Tang, Yan Zhao, Na Li, Feng Liu

**Affiliations:** a Beijing National Laboratory for Molecular Sciences , Key Laboratory of Bioorganic Chemistry and Molecular Engineering of Ministry of Education , College of Chemistry and Molecular Engineering , Peking University , Beijing 100871 , China . Email: liufeng@pku.edu.cn; b Institute of Materials , China Academy of Engineering Physics , Mianyang , 621700 , China

## Abstract

Reliably distinguishing single-nucleotide mutations (SNMs) at low abundance is of great significance in clinical diagnosis. However, the specificity of most current SNM discrimination methods based on the Watson–Crick hybridization is seriously limited by the cross-reactivity of the probe with closely related unintended sequences. Herein, we propose a sequestration-assisted molecular beacon (MB) strategy for highly specific SNM discrimination. The new SNM discrimination system consists of a target-specific MB and a series of hairpin sequestering agents (SEQs). The rationally designed hairpin SEQs can effectively sequester the corresponding unintended sequences and thus dramatically improve the hybridization specificity of the MB in recognizing SNMs. The developed SNM discrimination method shows remarkably high specificity (discrimination factors ranging from 12 to 1144 with a median of 117) against 20 model SNMs, and can work rapidly and robustly over a wide range of conditions. Notably, our SNM discrimination method can be easily combined with PCR amplification for the detection of *KRAS* G12D (c.35G>A) and G12V (c.35G>T) mutations at abundance as low as 0.5%. This work expands the rule set of designing hybridization-based SNM discrimination strategies and shows promising potential application in clinical diagnosis.

## Abstract 2

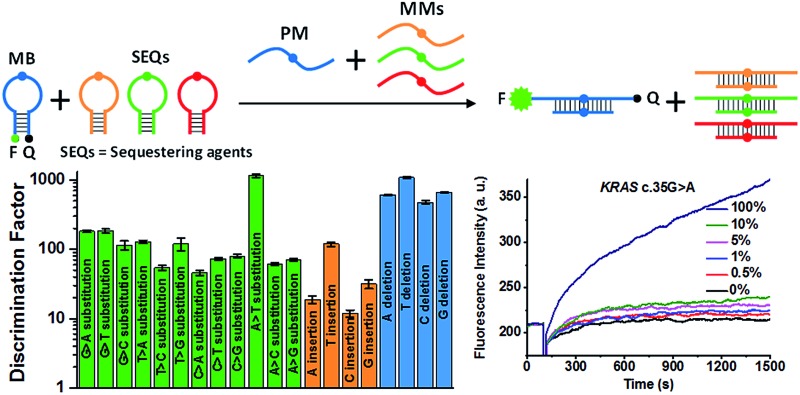
A sequestration-assisted molecular beacon strategy was proposed for highly specific discrimination and detection of single-nucleotide mutations at low abundance.

## Introduction

Single-nucleotide mutations (SNMs), including single-nucleotide substitutions, insertions and deletions, are important biomarkers for human diseases and drug resistance.^1,2^ Various technology platforms^3^ have been applied to the discrimination and detection of low abundance SNMs by using synthetic nucleic acid primers and probes, for example, through polymerase chain reaction (PCR),^4,5^ next-generation sequencing,^6,7^ microarrays^8,9^ and fluorescent *in situ* hybridization.^10,11^ Crucially, the discrimination ability of all these technologies and methods relies on the specificity of Watson–Crick base pairing at some step of their workflow, which is limited by the small difference in thermodynamic stability caused by a single-base mismatch.^12^ Many efforts have been devoted to the improvement of the discrimination ability by using denaturation approaches,^13,14^ enzymatic methods,^15–26^ synthetic nucleotide analogues^27,28^ and rationally designed probes such as molecular beacons,^29–32^ binary probes,^33,34^ triple-stem probes^35,36^ and toehold probes.^37–42^ However, most of these efforts have focused on the optimization of reaction condition and probe or primer design, and the discrimination ability is still seriously limited by the cross-reactivity with closely related unintended sequences. Recently, several competitive systems using the sequence-specific DNA sinks,^3,43^ the controller DNAs,^44^ the DNA-blocker strand^45^ and the peptide nucleic acid clamps^46^ were designed for SNM discrimination. These advances effectively improved the discrimination ability but suffered from complexity and required stringent condition control. As a result, it remains a major challenge to develop simple, robust and highly specific hybridization-based SNM discrimination strategies.

Herein, for the first time, we propose a sequestration-assisted molecular beacon (MB) strategy for highly specific SNM discrimination in homogeneous solutions. The new SNM discrimination system consists of a target-specific MB and a series of hairpin sequestering agents (SEQs). The rationally designed hairpin SEQs can sequester the closely related unintended sequences and thus effectively eliminate the cross-reactivity. By using fluorescence measurements, we quantitatively evaluated the discrimination ability of the developed SNM discrimination method against a series of SNMs, and also investigated the superiority of the hairpin SEQ as well as the condition robustness of the developed SNM discrimination method. Moreover, we explored the feasibility of combining our SNM discrimination method with PCR amplification for the detection of *KRAS* G12D (c.35G>A) and G12V (c.35G>T) mutations at low abundance to demonstrate the potential application in clinical diagnosis.

## Results and discussion

**Fig. 1:**
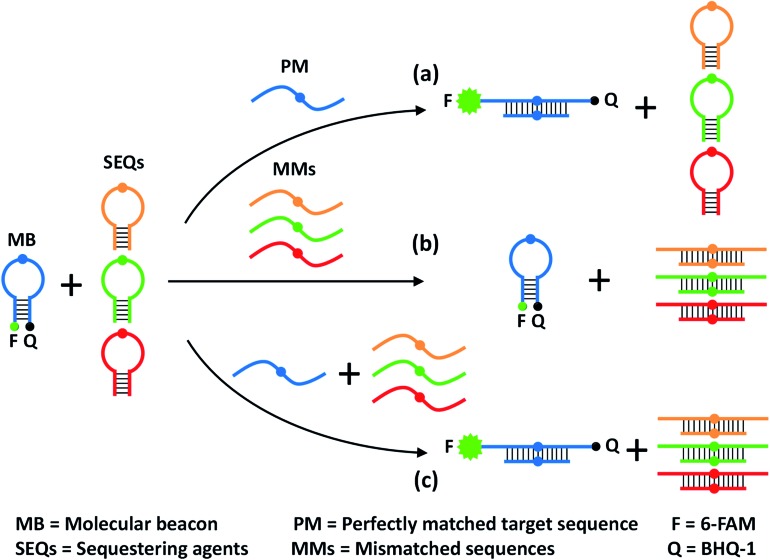
Schematic representation of the proposed sequestration-assisted MB strategy for SNM discrimination.

The design principle of the proposed sequestration-assisted MB strategy is illustrated in Fig. 1. The SNM discrimination system consists of a target-specific MB and a series of specific hairpin SEQs which can sequester the corresponding single-base mismatched sequences (MMs). The loop portion of the MB serves as a probe sequence that is perfectly complementary to the target sequence, and the stem of the MB is formed by two complementary 5-nt arm sequences with a fluorophore (F) and a quencher (Q) attached to the ends of the two arms respectively. The structure of the hairpin SEQs is similar to that of the MB, except that the hairpin SEQs have no fluorophore or quencher, and their loop sequences are complementary to the corresponding MMs. In the absence of the perfectly matched target sequence (PM), the 6-carboxyfluorescein (6-FAM) at the 5′ end of the MB is quenched by the black hole quenchers-1 (BHQ-1) at the 3′ end. After the addition of the PM, the MB hybridizes with the PM regardless of the SEQs, and the fluorescence signal is recovered because the unfolding of MB increases the spatial distance of 6-FAM and BHQ-1 (pathway a). In the presence of MMs only, a large excess of hairpin SEQs almost completely sequesters the MMs, so the MB still keeps the closed state (pathway b). When the PM and the MMs are present simultaneously, the MB can still be opened by the binding of PM to restore the fluorescence, and the MMs are also sequestered by the hairpin SEQs (pathway c). It is noteworthy that the sequestration-assisted MB strategy presented here (1) is developed from the classic MB system^47^ and is quite simple and enzyme-free, (2) can effectively eliminate the cross-reactivity of the MB with MMs by using hairpin SEQs, and (3) can be applied to the discrimination of other SNMs by easily altering the loop sequences of the MB and the hairpin SEQs.

**Fig. 2:**
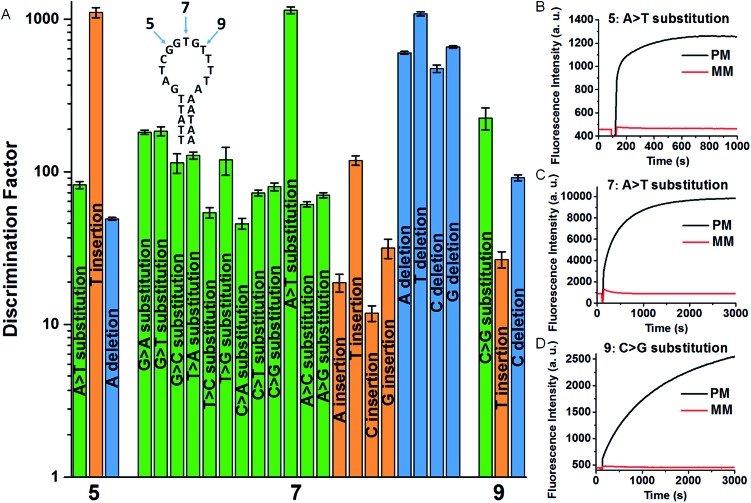
(A) DFs for the SNMs at the position 5, 7 and 9. The corresponding real-time fluorescence responses are shown in Fig. S1 and S2.† The inset shows the MB sequence and three positions of the corresponding SNMs. Real-time fluorescence responses of our SNM discrimination system to PM/MM pairs of A > T substitution at the position 5 (B) and 7 (C), and C > G substitution at the position 9 (D). The concentrations of MB, SEQ, PM and MM are 20, 800, 20 and 20 nM, respectively. The error bars represent the standard deviation of three measurements.

To quantitatively evaluate the discrimination ability of the developed SNM discrimination method, we performed time-resolved fluorescence measurements to get discrimination factors (DFs) of a series of SNMs at three different positions. The inset of Fig. 2A shows the corresponding locations of these positions in the MB. The DF is defined as the ratio of the net fluorescence intensity gain obtained with the PM to that obtained with the MM under the same conditions (DF = Δ*F*_PM_/Δ*F*_MM_). Thus, a larger DF value is indicative of greater specificity. To systematically investigate the specificity of our SNM discrimination method, we calculated the DFs of all possible 20 SNMs (including 12 substitutions, 4 insertions and 4 deletions) at the position 7. The oligonucleotide sequences of all strands are provided in Tables S1 and S2.† As can be seen from Fig. 2A, our strategy shows excellent discrimination ability with remarkable DF values ranging from 12 to 1144 with a median of 117, which is better than that of most SNM discrimination methods reported recently (Table S3†). To demonstrate the versatility of our method, we tested two additional positions (5 and 9). For SNMs at the position 5 and 9, the DFs of six representative SNMs are in the range from 27 to 1105 (Fig. 2A), indicating that our method is reliable for the discrimination of mutations at different positions. The real-time fluorescence responses to three representative PM/MM pairs at the position 5, 7 and 9 are shown in Fig. 2B–D. The above results indicate excellent discrimination ability of our SNM discrimination method, which is attributed to the following factors: (1) the MB itself provides a competing reaction for the probe–target hybridization, which possesses good specificity, (2) the MMs are sequestered by the rationally designed hairpin SEQs, thus non-specific hybridization of MMs with the MB is effectively eliminated, and (3) the hairpin structure of the SEQ increases the sequestration specificity, leading to further improvement in discrimination ability. These experimental results clearly demonstrate the remarkable discrimination ability of our SNM discrimination method.

**Fig. 3:**
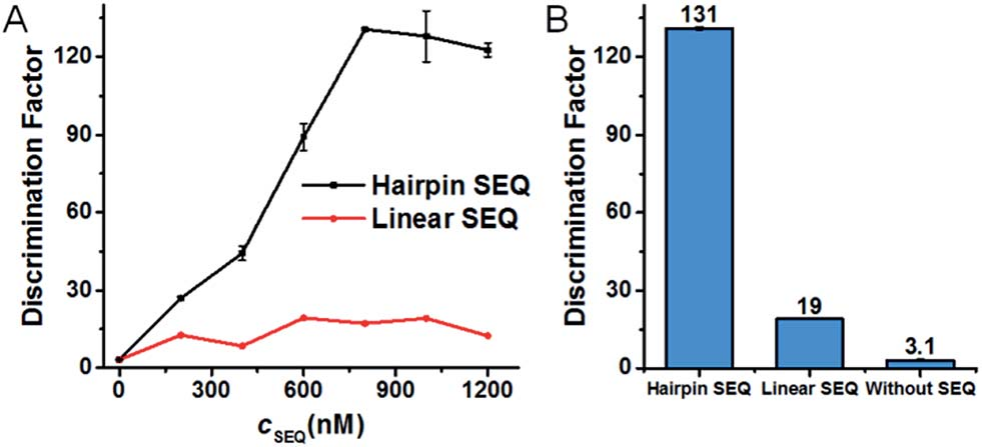
(A) DFs of SNM discrimination systems with different concentrations of the hairpin SEQ and the linear SEQ. (B) Maximum DFs of the SNM discrimination systems with the hairpin SEQ, with the linear SEQ and without the SEQ, respectively. The concentrations of all MB, PM and MM are 20 nM respectively. The error bars represent the standard deviation of three measurements.

According to the design rationale of the proposed discrimination strategy, the structure and concentration of the SEQ are considered as crucial factors for remarkably improving the SNM discrimination ability. To prove the superiority of the hairpin SEQ, we compared the specificity of three SNM discrimination systems with the hairpin SEQ, with the linear SEQ and without the SEQ, respectively. We performed fluorescence measurements and calculated the DFs of these SNM discrimination systems using G > T substitution at the position 7 as a model. As depicted in Fig. 3A, the maximum DF values 131, 19 and 3.1 are obtained with the hairpin SEQ, with the linear SEQ and without the SEQ (*i.e. c*_SEQ_ = 0), respectively. The SNM discrimination system with 800 nM hairpin SEQ achieves the highest DF and shows about 7-fold and 42-fold improvements compared to the SNM discrimination systems with the linear SEQ and without the SEQ respectively (Fig. 3B), clearly demonstrating the significant contribution of the hairpin SEQ to the discrimination ability. We also investigated the sensitivity of the proposed SNM discrimination system. The detection limits of our system and the simple MB system are 0.98 nM and 0.44 nM calculated by the 3S/N method from the corresponding linear relationships (Fig. S3†), respectively. Our method caused a slight reduction (about 2-fold) in sensitivity, due to the possible cross-reactivity of the SEQs with the PM. The results confirm that the rationally designed hairpin SEQ plays a key role in the dramatically enhanced specificity of the proposed sequestration-assisted MB strategy.

**Fig. 4:**
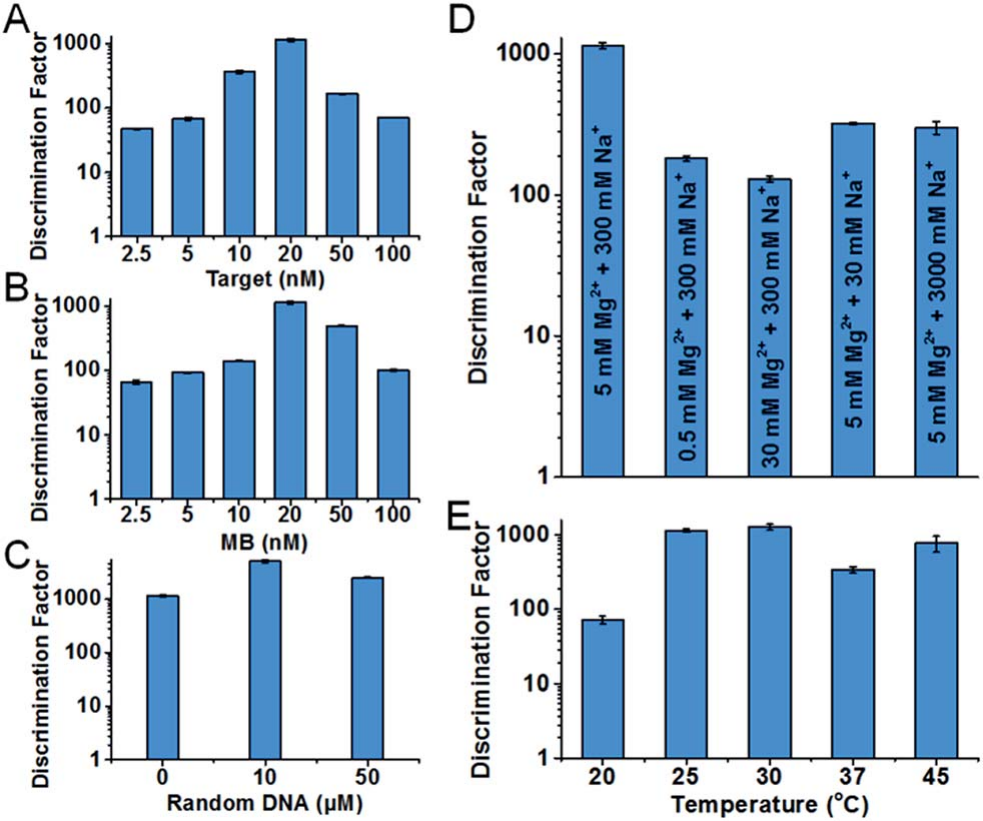
Characterization of the condition robustness of the developed SNM discrimination method, in different concentrations of the target (A), the MB (B), 50-nt random DNA sequences (C), in different salinity buffers (D) and at different temperatures (E). The error bars represent the standard deviation of three measurements. All of the corresponding real-time fluorescence responses are shown in Fig. S4–S8.†

Condition robustness and fast discrimination are key factors of SNM discrimination methods for the potential application in clinical diagnosis. We first investigated the robustness of the developed SNM discrimination method (using A > T substitution at the position 7 as a model) by obtaining DFs in a wide range of conditions. The results are shown in Fig. 4. When the concentrations of the target or the MB are changed in the range of 2.5–100 nM, the DFs remain greater than 47 and 66 respectively (Fig. 4A and B), implying that our SNM discrimination method can maintain good specificity in a wide concentration range of the target or the MB. The remarkable DFs (≥1144) are produced in up to 50 μM of 50-nt random DNA sequences (Fig. 4C), and the DFs are greater than 140 in buffers with different concentrations of Mg^2+^ and Na^+^ (0.5–50 mM Mg^2+^ and 30–3000 mM Na^+^, Fig. 4D). Therefore, biological samples or PCR products might be analyzed directly without purification or buffer-exchange procedures. Our SNM discrimination method is also robust throughout the temperature range of 20–45 °C with the corresponding DFs no less than 72 (Fig. 4E), meaning precise temperature-control equipment is not required. We next discussed how quickly our SNM discrimination method could distinguish PM/MM pairs. For all possible 20 SNMs at the position 7, a median DF of 76 can be achieved in only 10 minutes after the initiation of the reaction (Table S4†). The experiment results demonstrate that our highly specific SNM discrimination method (1) can work robustly over a wide range of temperatures, salinities, target/MB concentrations and in the presence of high concentration of 50-nt random DNA sequences, and (2) is capable of quickly distinguishing SNMs, thus having great potential to detect disease-related SNMs in biological samples.

**Fig. 5:**
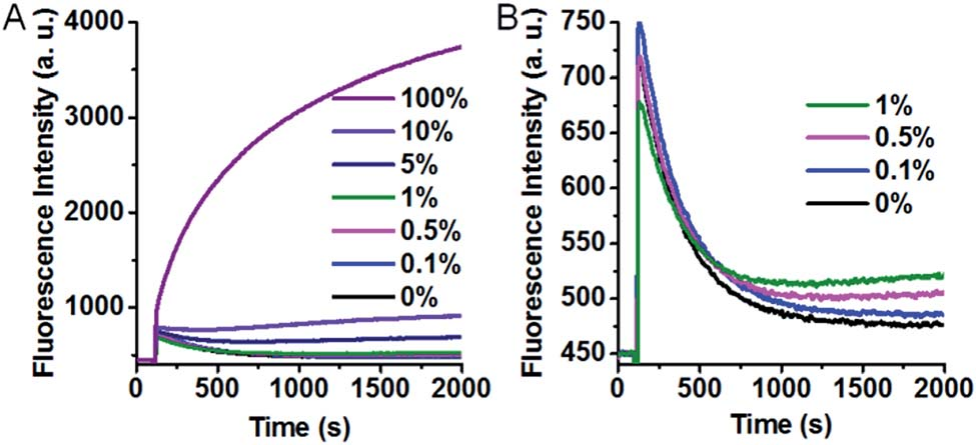
Real-time fluorescence responses of our SNM discrimination system to the PM at different abundances (from 0% to 100%) (A) and at low abundances (0–1%) in an enlarged scale (B). 100% means the tested sequences are all the PM. 0% means the tested sequences are all the MM.

The excellent specificity makes our SNM discrimination method well-suited for the detection of low abundance mutations. To evaluate this potential, we used A > T substitution at the position 7 as a model and measured the fluorescence responses of our SNM discrimination system to the PM at different abundances. As can be seen from Fig. 5A, the fluorescence intensity shows a gradual rise with the increasing PM percentage. An obvious fluorescence intensity increase can still be seen when only 0.1% PM is present (Fig. 5B), indicating that the PM can be successfully identified at abundance as low as 0.1%. The results well demonstrate the capability of our assay in detecting low abundance mutations.

**Fig. 6:**
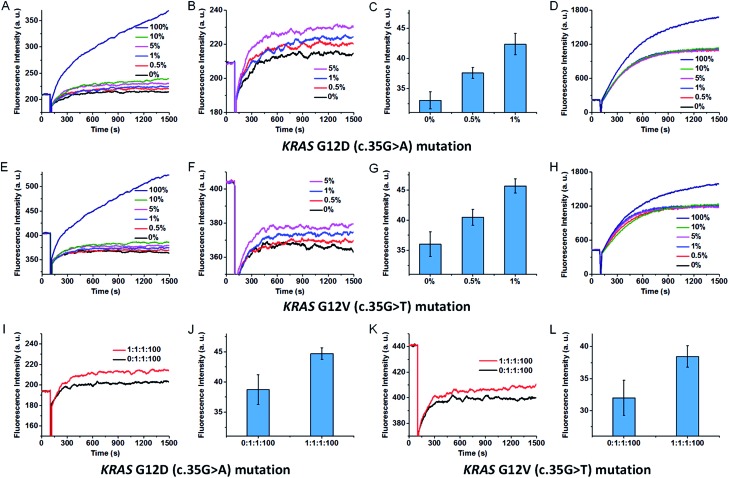
Real-time fluorescence responses of our SNM discrimination system in the detection of *KRAS* G12D (c.35G>A) mutation after PCR amplification at different abundances (from 0% to 100%) (A), and at low abundances (0–5%) in an enlarged scale (B). (C) Histograms showing the capacity of our method in the detection of low abundance *KRAS* G12D mutation. (D) Real-time fluorescence responses of classic MB system in the detection of *KRAS* G12D mutation after PCR amplification at different abundances (from 0% to 100%). (E)–(H) The corresponding experimental results in the detection of *KRAS* G12V (C.35G>T) mutation. 100% means the tested sequences are all mutant-type (mutant A or T). 0% means the tested sequences are all wild-type. (I)–(L) Detection of *KRAS* G12D (c.35G>A) and G12V (c.35G>T) mutations among a large excess of wild-type and the other two unintended mutants by combining our method with PCR amplification. (I) and (K) Real-time fluorescence responses. (J) and (L) Histograms. The error bars represent the standard deviation of three measurements.

Mutated *KRAS* genes are associated with lung cancer, colorectal cancer, ovarian cancer and pancreatic cancer.^46,48^ To further prove the potential application of our SNM discrimination method in clinical diagnosis, we combined this SNM discrimination method with PCR amplification to detect *KRAS* G12D (c.35G>A) and G12V (c.35G>T) mutations. We first performed the detection of *KRAS* G12D (c.35G>A) mutation. The mutant-type (mutant A) and wild-type sequences were mixed at 0 : 100, 0.5 : 99.5, 1 : 99, 5 : 95, 10 : 90 and 100 : 0 ratios to total concentrations of 0.5 pg μl^–1^, and amplified by asymmetric PCR to generate single-stranded amplicons. Two synthetic oligonucleotides were added to the PCR amplicons to unwind the secondary structure, and then the amplicons were analysed by our SNM discrimination method. As shown in Fig. 6A–C, the mutant-type target can be successfully identified at abundance as low as 0.5% in the presence of wild-type strands. In contrast, the classic MB systems (without the SEQs) could hardly distinguish and detect this mutation even at 10% abundance (Fig. 6D). The results demonstrate the capability of our assay in specific and sensitive detection of low abundance SNMs. Besides the wild-type sequence, other mutant-type sequences may also interfere with the detection of the exact mutant-type of interest. So we mixed the intended mutant-type (mutant A) with same amounts of the other two unintended mutant-types (mutant T and mutant C) and 100-fold excess of wild-type. After PCR amplification, three SEQs were added to simultaneously sequester the wild-type as well as the other two unintended mutants. As can be seen from Fig. 6I and J, the mixtures of three mutant-types and the wild-type at the ratio of mutant A : mutant T : mutant C : wild-type = 0 : 1 : 1 : 100 and 1 : 1 : 1 : 100 are successfully discriminated. To demonstrate the versatility of our approach, we also tested the proposed method against *KRAS* G12V (c.35G>T) mutation. The results are similar to that of the *KRAS* G12D (c.35G>A) mutation (shown in Fig. 6E–H, K and L). The results indicate that our sequestration-assisted MB strategy is versatile and can be applied to the detection of low abundance SNMs in PCR amplicons with high specificity, thus holding great potential for clinical application.

## Conclusions

In summary, we have successfully developed a simple and robust SNM discrimination method with remarkably high specificity using the sequestration-assisted MB strategy. The crucial element of the proposed strategy is the rationally designed hairpin SEQs that can effectively sequester the closely related unintended sequences and thus dramatically improve the hybridization specificity of the MB in recognizing SNMs. Our SNM discrimination method can work rapidly and robustly over a wide range of conditions and can be easily combined with PCR amplification to detect *KRAS* G12D (c.35G>A) and G12V (c.35G>T) mutations at low abundance, demonstrating the capability of our assay in specific and sensitive detection of low abundance SNMs. Moreover, the proposed strategy provides a general SNM discrimination method through simply altering the loop sequences of the MB and SEQs. We anticipate that this work offers a new route to design SNM discrimination strategies for clinical application.

## References

[cit1] Forbes S. A., Bindal N., Bamford S., Cole C., Kok C. Y., Beare D., Jia M., Shepherd R., Leung K., Menzies A., Teague J. W., Campbell P. J., Stratton M. R., Futreal P. A. (2011). Nucleic Acids Res..

[cit2] Loeb L. A. (2011). Nat. Rev. Cancer.

[cit3] Wang J. S., Zhang D. Y. (2015). Nat. Chem..

[cit4] Wu D. Y., Ugozzoli L., Pal B. K., Wallace R. B. (1989). Proc. Natl. Acad. Sci. U. S. A..

[cit5] Vogelstein B., Kinzler K. W. (1999). Proc. Natl. Acad. Sci. U. S. A..

[cit6] Mardis E. R. (2008). Annu. Rev. Genomics Hum. Genet..

[cit7] Schmitt M. W., Kennedy S. R., Salk J. J., Fox E. J., Hiatt J. B., Loeb L. A. (2012). Proc. Natl. Acad. Sci. U. S. A..

[cit8] Schena M., Shalon D., Davis R. W., Brown P. O. (1995). Science.

[cit9] Gunderson K. L., Steemers F. J., Lee G., Mendoza L. G., Chee M. S. (2005). Nat. Genet..

[cit10] Pinkel D., Landegent J., Collins C., Fuscoe J., Segraves R., Lucas J., Gray J. (1988). Proc. Natl. Acad. Sci. U. S. A..

[cit11] Wallner G., Amann R., Beisker W. (1993). Cytometry.

[cit12] SantaLucia, Jr J., Hicks D. (2004). Annu. Rev. Biophys. Biomol. Struct..

[cit13] Howell W. M., Jobs M., Gyllensten U., Brookes A. J. (1999). Nat. Biotechnol..

[cit14] Wittwer C. T., Reed G. H., Gundry C. N., Vandersteen J. G., Pryor R. J. (2003). Clin. Chem..

[cit15] Landegren U., Kaiser R., Sanders J., Hood L. (1988). Science.

[cit16] Lizardi P. M., Huang X., Zhu Z., Bray-Ward P., Thomas D. C., Ward D. C. (1998). Nat. Genet..

[cit17] Lyamichev V., Mast A. L., Hall J. G., Prudent J. R., Kaiser M. W., Takova T., Kwiatkowski R. W., Sander T. J., de Arruda M., Arco D. A., Neri B. P., Brow M. A. D. (1999). Nat. Biotechnol..

[cit18] Dominguez P. L., Kolodney M. S. (2005). Oncogene.

[cit19] Li J., Wang L., Mamon H., Kulke M. H., Berbeco R., Makrigiorgos G. M. (2008). Nat. Med..

[cit20] Xiang Y., Lu Y. (2012). Anal. Chem..

[cit21] Jou A. F. J., Lu C. H., Ou Y. C., Wang S. S., Hsu S. L., Willner I., Ho J. A. A. (2015). Chem. Sci..

[cit22] Wu T., Xiao X., Zhang Z., Zhao M. (2015). Chem. Sci..

[cit23] Su F., Wang L., Sun Y., Liu C., Duan X., Li Z. (2015). Chem. Sci..

[cit24] Zhang H., Lai M., Zuehlke A., Peng H., Li X. F., Le X. C. (2015). Angew. Chem., Int. Ed..

[cit25] Xiao X., Wu T., Gu F., Zhao M. (2016). Chem. Sci..

[cit26] Xu Q., Huang S. Q., Ma F., Tang B., Zhang C. Y. (2016). Anal. Chem..

[cit27] Nielsen P. E., Egholm M., Berg R. H., Buchardt O. (1991). Science.

[cit28] Singh S. K., Nielsen P., Koshkin A. A., Wengel J. (1998). Chem. Commun..

[cit29] Tyagi S., Kramer F. R. (1996). Nat. Biotechnol..

[cit30] Tyagi S., Bratu D. P., Kramer F. R. (1998). Nat. Biotechnol..

[cit31] Tan W., Wang K., Drake T. J. (2004). Curr. Opin. Chem. Biol..

[cit32] Zheng J., Yang R., Shi M., Wu C., Fang X., Li Y., Li J., Tan W. (2015). Chem. Soc. Rev..

[cit33] Cardullo R. A., Agrawal S., Flores C., Zamecnik P. C., Wolf D. E. (1988). Proc. Natl. Acad. Sci. U. S. A..

[cit34] Kolpashchikov D. M. (2010). Chem. Rev..

[cit35] Xiao Y., Plakos K. J. I., Lou X., White R. J., Qian J., Plaxco K. W., Soh H. T. (2009). Angew. Chem., Int. Ed..

[cit36] Zhou H., Liu J., Xu J. J., Chen H. Y. (2011). Anal. Chem..

[cit37] Li Q., Luan G., Guo Q., Liang J. (2002). Nucleic Acids Res..

[cit38] Zhang Z., Zeng D., Ma H., Feng G., Hu J., He L., Li C., Fan C. (2010). Small.

[cit39] Wang D., Tang W., Wu X., Wang X., Chen G., Chen Q., Li N., Liu F. (2012). Anal. Chem..

[cit40] Zhang D. Y., Chen S. X., Yin P. (2012). Nat. Chem..

[cit41] Chen S. X., Zhang D. Y., Seelig G. (2013). Nat. Chem..

[cit42] Yao D., Song T., Sun X., Xiao S., Huang F., Liang H. (2015). J. Am. Chem. Soc..

[cit43] Chen S. X., Seelig G. (2016). J. Am. Chem. Soc..

[cit44] Sayyed D. R., Nimse S. B., Song K. S., Kim T. (2014). Chem. Commun..

[cit45] Wu T., Xiao X., Gu F., Zhao M. (2015). Chem. Commun..

[cit46] Das J., Ivanov I., Montermini L., Rak J., Sargent E. H., Kelley S. O. (2015). Nat. Chem..

[cit47] Ricci F., Vallée-Bélisle A., Plaxco K. W. (2011). PLoS Comput. Biol..

[cit48] Bryant K. L., Mancias J. D., Kimmelman A. C., Der C. J. (2014). Trends Biochem. Sci..

