# Supplementary material for: Ultra-specific discrimination of single-nucleotide mutations using sequestration-assisted molecular beacons
Source: Chem Sci. 2016 Sep 19;8(2):1021–6. doi: 10.1039/c6sc03048c (PMC5356502; doi:10.1039/c6sc03048c)
Supplement: Supplementary file 1 [file SC-008-C6SC03048C-s001.pdf]

Electronic Supplementary Material (ESI) for Chemical Science.
This journal is © The Royal Society of Chemistry 2016

Electronic Supplementary Information

Ultra-specific discrimination of single-nucleotide mutations using sequestration-assisted molecular beacons

Shichao Hu,‡[a] Wei Tang,‡[b] Yan Zhao,[a] Na Li[a] and Feng Liu*[a]

[a]Beijing National Laboratory for Molecular Sciences, Key Laboratory of Bioorganic Chemistry and Molecular Engineering of Ministry of Education, College of Chemistry and Molecular Engineering, Peking University, Beijing 100871, China.

[b]Institute of Materials, China Academy of Engineering Physics, Mianyang, 621700, China.

‡ These authors contributed equally.

* Corresponding author. E-mail: liufeng@pku.edu.cn

Materials and Reagents

All HPLC-purified DNA oligonucleotides were purchased from Sangon Inc. (Shanghai, China). 2×PCR Master Mix was purchased from BioTeke Inc. (Beijing, China). The DNA sequences and modifications are listed in Table S1 and S2. The DNA samples were dissolved in TE buffer solution (50 mM tris(hydroxymethyl)aminomethane (Tris) and 1 mM ethylenediaminetetraacetic acid (EDTA); pH 8.0) and stored in the dark at 4 °C. Deionized water was used in all experiments.

Fluorescence Measurements and Data Processing

Fluorescence measurements were performed by using an F-7000 fluorescence spectrophotometer (Hitachi, Japan) with a water-bath circulator to maintain the temperature. Sample solutions were excited at 495 nm with a slit size of 10 nm, and the emission signal was recorded at 520 nm with a slit size of 10 nm or 5 nm (5 nm was used when the fluorescence signal is so large to cause photo detector saturation).

For preparation of the mixed solution containing MB and SEQ, the MB was mixed with the SEQ in TNM buffer solution (50 mM Tris, 100 mM NaCl and 5 mM $MgCl_2$; pH 7.5) at final concentrations of 20 nM MB and 800 nM SEQ. The resulting solutions were heated at 95 °C for 5 min and then slowly cooled down to room temperature over 50 minutes. 300 μL mixed solution containing MB and SEQ was

used in each measurement.

The mixed solution containing MB and SEQ in the cuvette was first incubated in the F-7000 fluorescence spectrophotometer for about 10 minutes to allow temperature equilibration, and the fluorescence data was recorded for 100 seconds to obtain the background fluorescence. Afterwards, 2 μL target solution was added into the cuvette and mixed quickly within 20 s, then the fluorescence data was continuously recorded until the fluorescence signal reached a relatively steady state. The DFs were calculated using the equation DF=$\Delta F_{PM}/\Delta F_{MM}$. ΔF_{PM} and ΔF_{MM} represent the net fluorescence intensity gains obtained with PM and MM, respectively.

Detection of *KRAS* Mutation in PCR Amplicons

To a 200 μL PCR tube, 44 μL water, 50 μL 2×PCR Master Mix, 2 μL forward primers (0.8 pmol), 2 μL reverse primers (40 pmol), and 2 μL mixed ssDNA templates (total amount 2 fmol, 53 pg) (see Table S2 for DNA oligonucleotide sequences) were added and mixed well. PCR procedure (94.0 ^{o}C for 30 s, 57.5 ^{o}C for 20 s, 72.0 ^{o}C for 30 s, 30 cycles) was performed by using a DNA Engine Opticon 2 (MJ Research, USA). After the PCR amplification, 3 μL (60 pmol) Opener1 and Opener2 respectively were added to the amplicons to unwind the secondary structure. The fluorescence data was measured following the same procedure described in "Fluorescence Measurements and Data Processing" except using 100 μL of PCR products after the addition of Opener1 and Opener2 instead of 2 μL target solution.

Table S1 DNA oligonucleotide sequences used in exploring performance of SNM discrimination systems.

Name	Sequence (from 5' to 3')
MB-7A	FAM-A-TTATT-GATCGGAGTTTTA-AATAA-G-BHQ
MB-7T	FAM-A-TTATT-GATCGGTGTTTTA-AATAA-G-BHQ
MB-7C	FAM-A-TTATT-GATCGGCGTTTTA-AATAA-G-BHQ
MB-7G	FAM-A-TTATT-GATCGGGGTTTTA-AATAA-G-BHQ
MB-5T	FAM-A-TTATT-GATCGGCGTTTTA-AATAA-G-BHQ
MB-9G	FAM-A-TTATT-GATCGGCGTTTTA-AATAA-G-BHQ
SEQ-7A	A-CTCTT-GATCGGAGTTTTA-AAGAG-G
SEQ-7T	A-CTCTT-GATCGGTGTTTTA-AAGAG-G
SEQ-7C	A-CTCTT-GATCGGCGTTTTA-AAGAG-G
SEQ-7G	A-CTCTT-GATCGGGGTTTTA-AAGAG-G
SEQ-7IA	A-CTCTT-GATCGGCAGTTTTA-AAGAG-G
SEQ-7IT	A-CTCTT-GATCGGCTGTTTTA-AAGAG-G
SEQ-7IC	A-CTCTT-GATCGGCCGTTTTA-AAGAG-G
SEQ-7IG	A-CTCTT-GATCGGCGGTTTTA-AAGAG-G
SEQ-7D	A-CTCTT-GATCGGGTTTTA-AAGAG-G
SEQ-5A	A-CTCTT-GATCGGCGATTTA-AAGAG-G
SEQ-5IA	A-CTCTT-GATCGGCGTATTTA-AAGAG-G
SEQ-5D	A-CTCTT-GATCGGCGTTTA-AAGAG-G
SEQ-9C	A-TTATT-GATCCGCGTTTTA-AATAA-G
SEQ-9IA	A-CTCTT-GATCGAGCGTTTTA-AAGAG-G
SEQ-9D	A-TTATT-GATCGCGTTTTA-AATAA-G
Target-7A	TAAAACACCGATC
Target-7T	TAAAACTCCGATC
Target-7C	TAAAACCCCGATC
Target-7G	TAAAACGCCGATC
Target-7IA	TAAAACAGCCGATC
Target-7IT	TAAAACTGCCGATC
Target-7IC	TAAAACCGCCGATC
Target-7IG	TAAAACGGCCGATC
Target-7D	TAAAACCCGATC
Target-5A	TAAAACGCCGATC
Target-5T	TAAATCGCCGATC
Target-5IT	TAAATACGCCGATC
Target-5D	TAAACGCCGATC
Target-9C	TAAAACGCCGATC
Target-9G	TAAAACGCGGATC
Target-9IT	TAAAACGCTCGATC
Target-9D	TAAAACGCGATC

Table S2 DNA oligonucleotide sequences used in the detection of *KRAS* G12D (c.35G>A) and G12V (c.35G>T) mutations.

Name	Sequence (from 5’ to 3’)
MB-c.35G>A	FAM-A-TTATT-CCTACGCCATCAG-AATAA-G-BHQ
MB-c.35G>T	FAM-A-TTATT-CCTACGCCAACAG-AATAA-G-BHQ
SEQ-wild-type	A-CTCTT-CCTACGCCACCAG-AAGAG-G
SEQ-c.35G>A	A-CTCTT-CCTACGCCATCAG-AAGAG-G
SEQ-c.35G>T	A-CTCTT-CCTACGCCAACAG-AAGAG-G
SEQ-c.35G>C	A-CTCTT-CCTACGCCAGCAG-AAGAG-G
Wild-type	GCCTGCTGAAAATGACTGAATATAAACTTGTGGTAGT TGGAGCTGGTGGCGTAGGCAAGAGTGCCTTGACGAT ACAGCTAA
Mutant A	GCCTGCTGAAAATGACTGAATATAAACTTGTGGTAGT TGGAGCTGATGGCGTAGGCAAGAGTGCCTTGACGAT ACAGCTAA
Mutant T	GCCTGCTGAAAATGACTGAATATAAACTTGTGGTAGT TGGAGCTGTTGGCGTAGGCAAGAGTGCCTTGACGAT ACAGCTAA
Mutant C	GCCTGCTGAAAATGACTGAATATAAACTTGTGGTAGT TGGAGCTGCTGGCGTAGGCAAGAGTGCCTTGACGAT ACAGCTAA
Forward primer	TTAGCTGTATCGTCAAGGCACTC
Reverse primer	GCCTGCTGAAAATGACTGAATATA
Opener1	CTCCAACTACCACAAGTTTATATTCAGTCATTTTCAG CAGGC
Opener2	TTAGCTGTATCGTCAAGGCACTCTTG

Table S3 Comparison between the proposed sequestration-assisted MB strategy with other SNM discrimination approaches reported since 2014.[a]

Discrimination format	Discrimination Factor (DF)	Reference
**Enzyme-free approach**		
Sequestration-assisted molecular beacon	12–1144, median=117 (20 SNMs)	This work
Discrimination by competition and catalytic amplification	23 (A>G substitution)	1
X-probe based toehold-mediated strand displacement	2.0–20[b] (11 SNMs)	2
Three-dimensional nanotip sensing array	26[b] (T>C substitution)	3
Piezoelectric plate sensor coated with a locked nucleic acid probe	~5.8 (A>C substitution)	4
Sandwich hybridization on magnetic beads	3.2 (G>A substitution)	5
Spontaneous cascade DNA branch migration	7.8–17.9, median=12 (3 SNMs)	6
Energy driven cascade recognition	45–109, median=70 (6 SNMs)	7
Gapped-duplex-based approach	62–225[b] (3 SNMs)	8
Enhanced solid-phase hybridization	5[b] (A>C substitution)	9
Switchable lanthanide luminescence binary probes	~0.9–1400, median between 10–20 (15 SNMs)	10
DNA–streptavidin dendrimer amplified sensing platform	17–72 (3 SNMs)	11
Enhanced destabilization of mismatched DNA using gold nanoparticles	3.8–5.2 (4 SNMs)	12
Simulation-guided DNA probe and sink design	Median β =890[c] (44 SNMs)	13
Protected DNA strand displacement	3.6–2×10^3, median=26.4 (12 SNMs)	14
DNA–gold nanoparticle probe-fueled DNA strand displacements	5.6–58[b] (18 SNMs)	15
Adenosine-based molecular beacon	7.5–10[b] (3 SNMs)	16
Locked nucleic acid-integrated with toehold-mediated strand displacement	29[b] (G>T substitution)	17
G-rich hairpin probes	1.7–8.9[b] (3 SNMs)	18
Blocker-enhanced hybridization	9.6–36[b] (6 SNMs)	19
Controller DNA technology	>100 (6 SNMs)	20
Gold nanoparticle-conducted DNA bioarrays	6.8–19.7 (12 SNMs)	21
Optimizing temperature hybridization	~1.4[b] (G>T substitution)	22
DNA-fueled molecular machine	68–137[b] (5 SNMs)	23
Enhanced DNA toehold exchange	14–42[b] (19 SNMs)	24

reaction		
**Enzyme approach**		
Controllable mismatched ligation	6.8–42[b] (12 SNMs)	25
Strand displacement and selective digestion	50.2–970, median=95.7 (15 SNMs)	26
Nicking endonuclease assisted target recycling and hyperbranched rolling circle amplification	~4.8 (C>A substitution)	27
5’-abasic lesion enhanced isothermal ligase chain reaction	7–12 (3 SNMs)	28
Ligation chain reaction	13[b] (C>A substitution)	29
Nicking endonuclease-assisted target recycling and magnetic nanoparticle separation	~4[b] (A>G substitution)	30
Abasic site modified fluorescent probe and lambda exonuclease	158–902, median=499 (9 SNMs)	31
Lambda exonuclease and a chemically modified DNA substrate structure	4.0–320, median=30 (12 SNMs)	32
T7 exonuclease digestion with target cyclic amplification	26 (C>T substitution)	33
Binding-induced DNA nanomachine	~5.9 (G>A substitution)	34
Specific amplification using variants of a *thermus aquaticus* DNA polymerase	1485 (G>A substitution)	35
Hybridization assay with ligation-mediated amplification	3.8–28[b] (4 SNMs)	36
Rolling circle amplification combined with gold nanoparticles-aptamer labeling	27 (G>C substitution)	37
G-quadruplex based two-stage isothermal exponential amplification	5.1[b] (C>G substitution)	38
Toehold-mediated strand displacement triggered isothermal DNA amplification	7.5–12.4 (3 SNMs)	39

[a]Not including the works from which we cannot obtain DFs.

[b]Calculated from data supplied in the articles.

[c]β is defined as the normalized fold-change, which is not identical to DF.

References

1 S. X. Chen and G. Seelig, *J. Am. Chem. Soc.*, 2016, **138**, 5076–5086.

2 X. Chen, D. Zhou, H. Shen, H. Chen, W. Feng and G. Xie, *Sci. Rep.*, 2016, **6**, 20257.

3 R. Esfandyarpour, L. Yang, Z. Koochak, J. S. Harris and R. W. Davis, *Biomed. Microdevices*, 2016, **18**, 7.

4 C. Kirimli, W. H. Shih and W. Y. Shih, *Analyst*, 2016, **141**, 1421–1433.

5 H. T. Ngo, N. Gandra, A. M. Fales, S. M. Taylor and T. Vo-Dinh, *Biosens. Bioelectron.*, 2016, **81**, 8–14.

6 T. Wang, L. Zhou, S. Bai, Z. Zhang, J. Li, X. Jing and G. Xie, *Biosens. Bioelectron.*, 2016, **78**,

464–470.
7 Z. Zhang, J. L. Li, J. Yao, T. Wang, D. Yin, Y. Xiang, Z. Chen and G. Xie, *Biosens. Bioelectron.*, 2016, **79**, 488–494.
8 R. E. Fernandez, S. E. Williams, R. Li and A. Zhou, *Electrochem. Commun.*, 2015, **56**, 1–5.
9 H. Joda, V. Beni, I. Katakis and C. K. O'Sullivan, *Anal. Biochem.*, 2015, **474**, 66–68.
10 U. Karhunen, E. Malmi, E. Brunet, J. C. Rodríguez-Ubis and T. Soukka, *Sens. Actuators B Chem.*, 2015, **211**, 297–302.
11 Y. Zhao, H. Wang, W. Tang, S. Hu, N. Li and F. Liu, *Chem. Commun.*, 2015, **51**, 10660–10663.
12 A. Sedighi, V. Whitehall and P. C. H. Li, *Nano. Res.*, 2015, **8**, 3922–3933.
13 J. S. Wang and D. Y. Zhang, *Nat. Chem.*, 2015, **7**, 545–553.
14 D. A. Khodakov, A. S. Khodakova, D. M. Huang, A. Linacre and A. V. Ellis, *Sci. Rep.*, 2015, **5**, 8721.
15 H. Li, S. Xiao, D. Yao, M. H. W. Lam and H. Liang, *Chem. Commun.*, 2015, **51**, 4670–4673.
16 T. E. Du, X. Mao, M. Y. Jin, T. Zhang and Y. Zhang, *Sens. Actuators B Chem.*, 2014, **198**, 194–200.
17 Z. F. Gao, Y. Ling, L. Lu, N. Y. Chen, H. Q. Luo and N. B. Li, *Anal. Chem.*, 2014, **86**, 2543–2548.
18 H. Li, Z. S. Wu, Z. Shen, G. Shen and R. Yu, *Nanoscale*, 2014, **6**, 2218–2227.
19 S. Okumura, R. Kuroda and K. Inouye, *Appl. Biochem. Biotechnol.*, 2014, **174**, 494–505.
20 D. R. Sayyed, S. B. Nimse, K. S. Song and T. Kim, *Chem. Commun.*, 2014, **50**, 12344–12347.
21 A. Sedighi and P. C. H. Li, *Anal. Biochem.*, 2014, **448**, 58–64.
22 S. N. Topkaya, B. Kosova and M. Ozsoz, *Clin. Chim. Acta*, 2014, **429**, 134–139.
23 T. Song, S. Xiao, D. Yao, F. Huang, M. Hu and H. Liang, *Adv. Mater.*, 2014, **26**, 6181–6185.
24 H. Xu, W. Deng, F. Huang, S. Xiao, G. Liu and H. Liang, *Chem. Commun.*, 2014, **50**, 14171–14174.
25 Q. Xu, S. Q. Huang, F. Ma, B. Tang and C. Y. Zhang, *Anal. Chem.*, 2016, **88**, 2431–2439.
26 Y. Yu, T. Wu, A. Johnson-Buck, L. Li and X. Su, *Biosens. Bioelectron.*, 2016, **82**, 248–254.
27 L. Yang, Y. Tao, G. Yue, R. Li, B. Qiu, L. Guo, Z. Lin and H. H. Yang, *Anal. Chem.*, 2016, **88**, 5097–5103.
28 A. Kausar, E. A. Osman, T. Gadzikwa and J. M. Gibbs-Davis, *Analyst*, 2016, **141**, 4272–4277.
29 S. Bi, Z. Zhang, Y. Dong and Z. Wang, *Biosens. Bioelectron.*, 2015, **65**, 139–144.
30 N. Li, Z. F. Gao, B. H. Kang, N. B. Li and H. Q. Luo, *RSC Adv.*, 2015, **5**, 20020–20024.
31 T. Wu, X. Xiao, F. Gu and M. Zhao, *Chem. Commun.*, 2015, **51**, 17402–17405.
32 T. Wu, X. Xiao, Z. Zhang and M. Zhao, *Chem. Sci.*, 2015, **6**, 1206–1211.
33 Z. K. Wu, D. M. Zhou, Z. Wu, X. Chu, R. Q. Yu and J. H. Jiang, *Chem. Commun.*, 2015, **51**, 2954–2956.
34 H. Zhang, M. Lai, A. Zuehlke, H. Peng, X. F. Li and X. C. Le, *Angew. Chem. Int. Ed.*, 2015, **54**, 14326–14330.
35 M. Drum, R. Kranaster, C. Ewald, R. Blasczyk and A. Marx, *Plos One*, 2014, **9**, e96640.
36 K. Brückner, K. Schwarz, S. Beck and M. W. Linscheid, *Anal. Chem.*, 2014, **86**, 585–591.
37 Y. He, D. Chen, M. Li, L. Fang, W. Yang, L. Xu and F. Fu, *Biosens. Bioelectron.*, 2014, **58**, 209–213.
38 J. Nie, D. W. Zhang, C. Tie, Y. L. Zhou and X. X. Zhang, *Biosens. Bioelectron.*, 2014, **56**,

237–242.
39 J. Zhu, Y. Ding, X. Liu, L. Wang and W. Jiang, *Biosens. Bioelectron.*, 2014, **59**, 276–281.

Table S4 Experimentally observed DFs for the SNMs at the position 7 in 10 minutes after the initiation of the reaction.

SNM type	Discrimination factor	SNM type	Discrimination factor
G>A substitution	139	A insertion	5
G>T substitution	142	T insertion	15
G>C substitution	116	C insertion	3
T>A substitution	122	G insertion	3
T>C substitution	70	A deletion	166
T>G substitution	102	T deletion	45
C>A substitution	14	C deletion	90
C>T substitution	21	G deletion	296
C>G substitution	40		
A>T substitution	198		
A>C substitution	81		
A>G substitution	26		

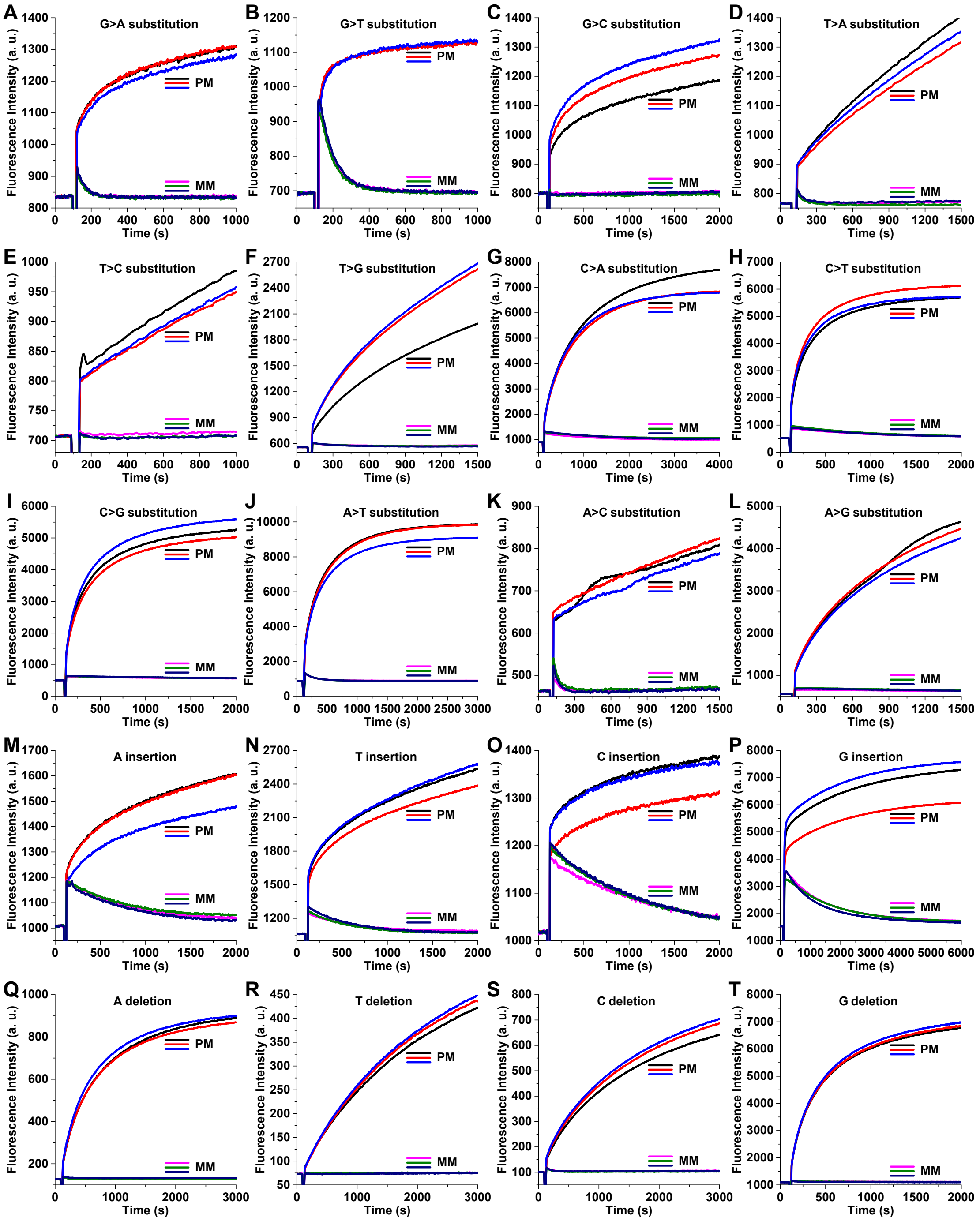

Fig. S1 Fluorescence responses of the developed SNM discrimination system to PM/MM pairs of all possible 20 SNMs at the position 7. The concentrations of all PMs and MMs were 20 nM. Each experiment was repeated three times.

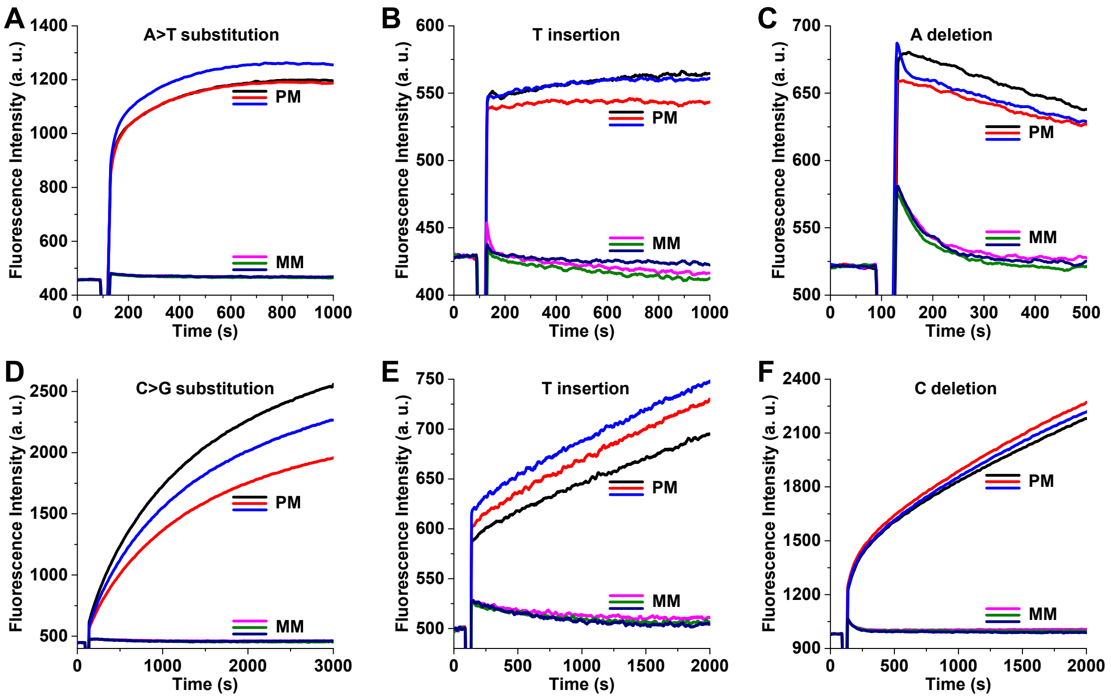

Fig. S2 Fluorescence responses of the developed SNM discrimination system to PM/MM pairs of six representative SNMs at the position 5 (A–C) and 9 (D–F). The concentrations of all PMs and MMs were 20 nM. Each experiment was repeated three times.

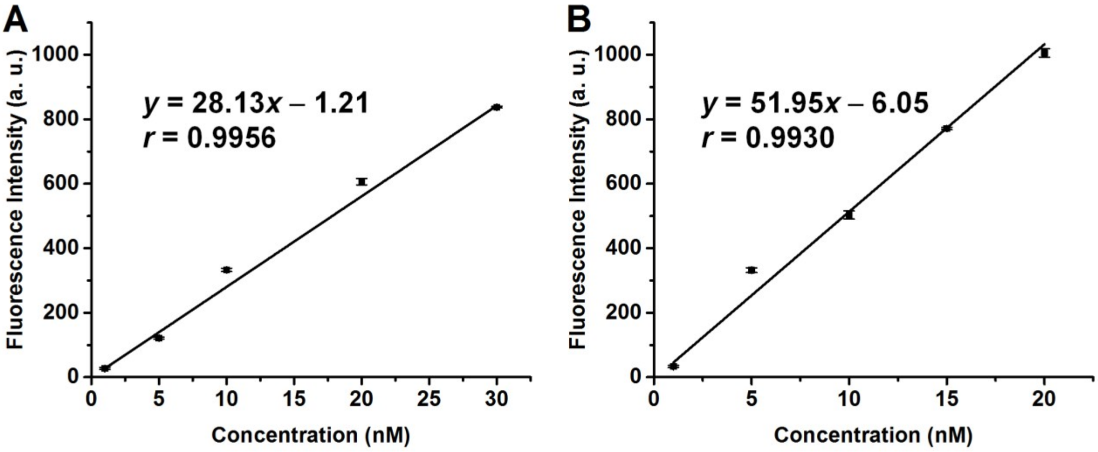

Fig. S3 Linear relationships between the fluorescence intensities and the PM of A>T substitution concentrations of the proposed SNM discrimination system (A) and the simple MB system (B). The concentrations of the MB and SEQ are 20 and 800 nM, respectively. The error bars are standard deviations of three repetitive measurements.

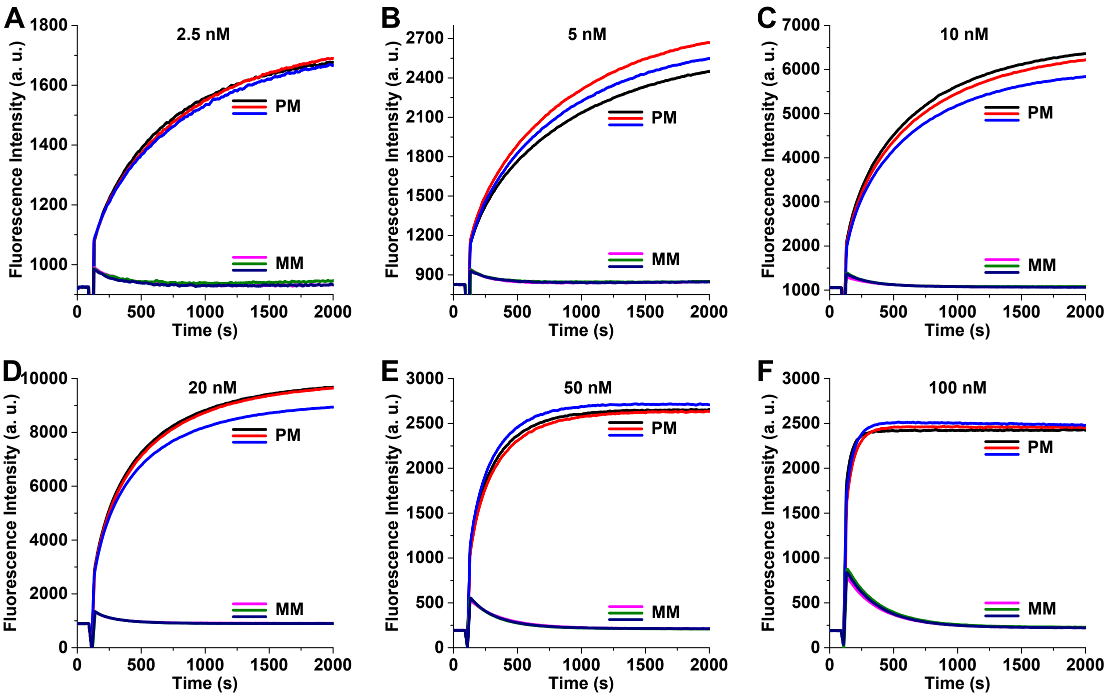

Fig. S4 Fluorescence responses of the developed SNM discrimination system to different concentrations of PM/MM pairs of A>T substitution at position 7. The concentrations of both PM and MM were the same. Each experiment was repeated three times.

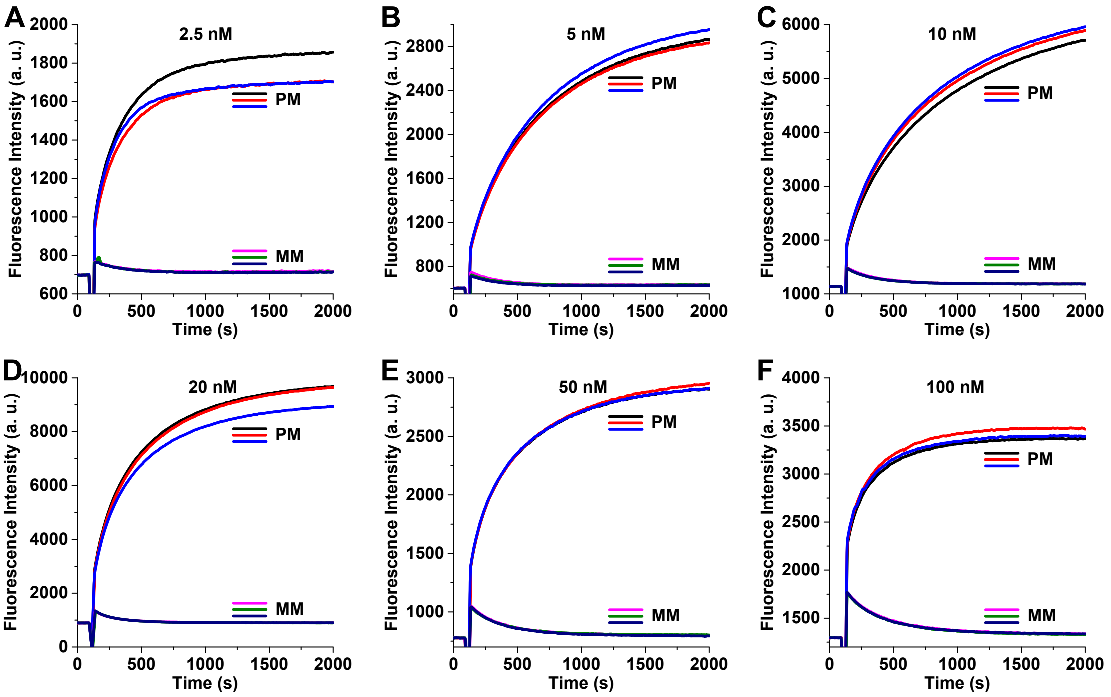

Fig. S5 Fluorescence responses of the developed SNM discrimination system using different concentrations of MBs to PM/MM pairs of A>T substitution at position 7. The concentrations of both PM and MM were 20 nM. Each experiment was repeated three times.

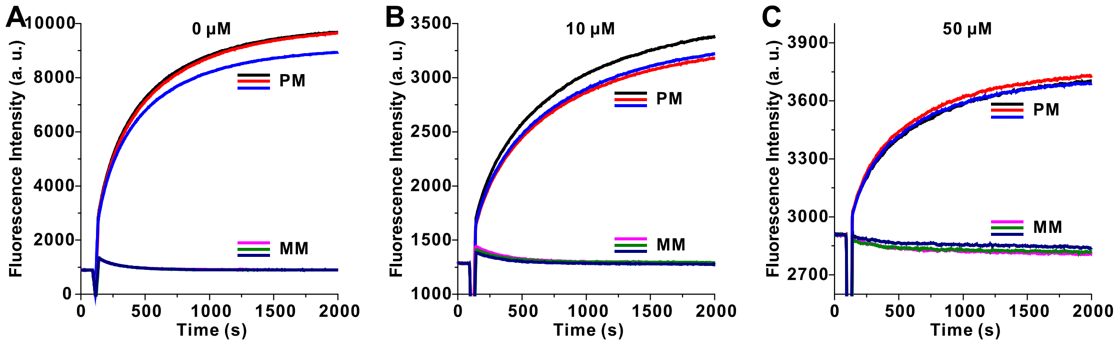

Fig. S6 Fluorescence responses of the developed SNM discrimination system to PM/MM pairs of A>T substitution at position 7 in the presence of different concentrations of 50-nt random DNA sequences. The concentrations of both PM and MM were 20 nM. Each experiment was repeated three times.

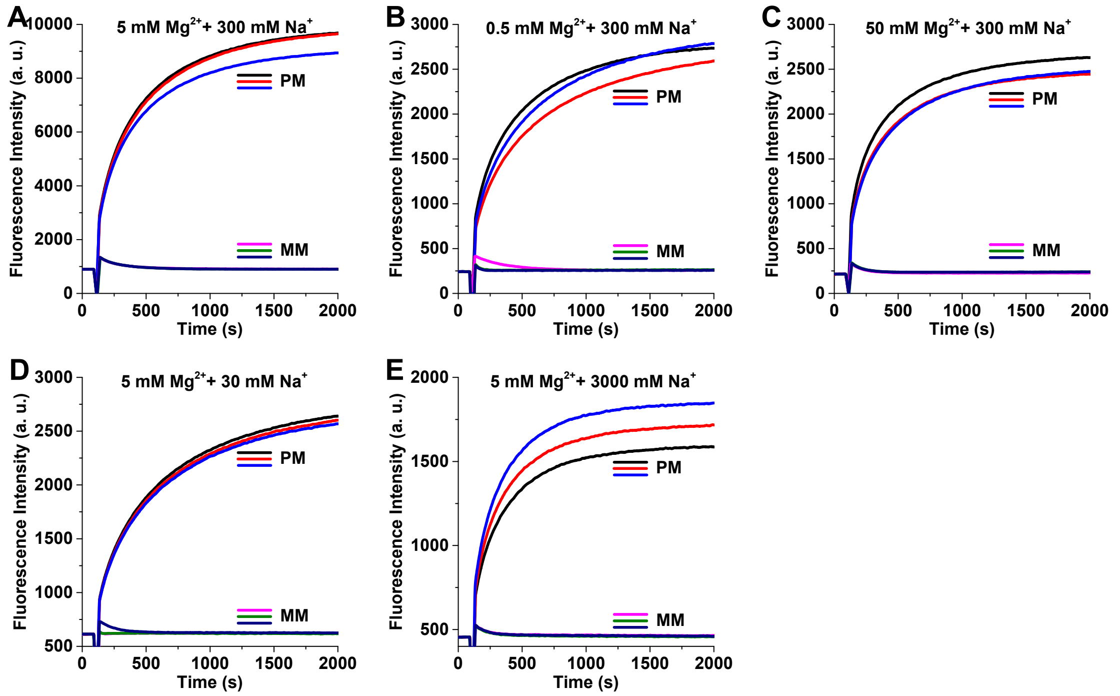

Fig. S7 Fluorescence responses of the developed SNM discrimination system to PM/MM pairs of A>T substitution at position 7 in different salinity buffers. The concentrations of both PM and MM were 20 nM. Each experiment was repeated three times.

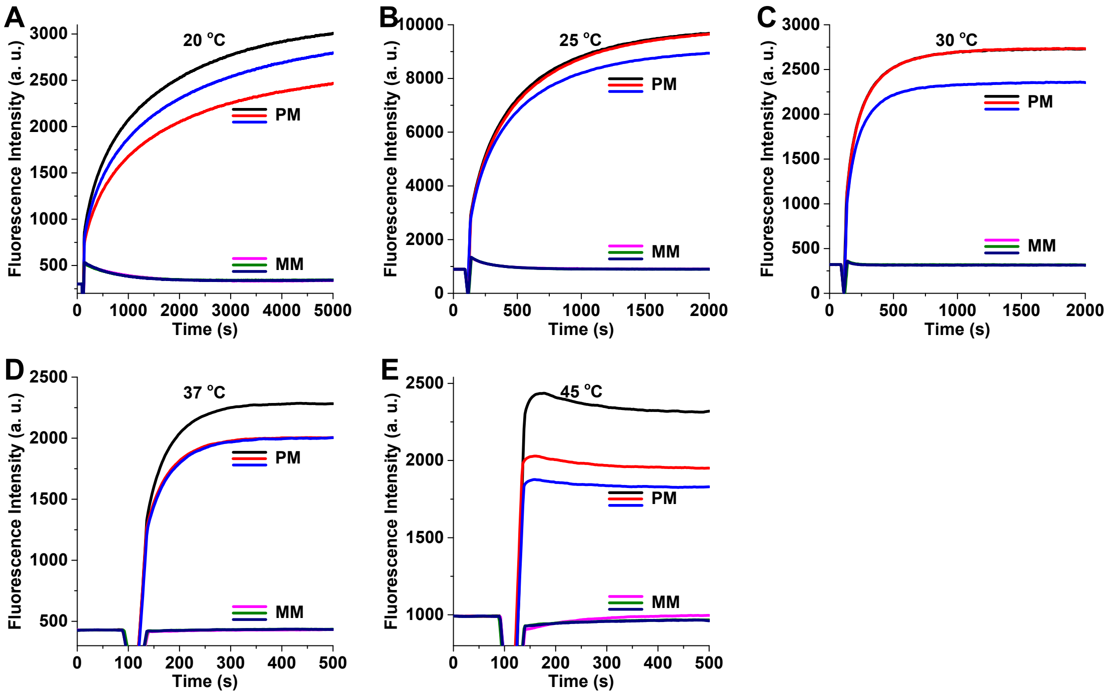

Fig. S8 Fluorescence responses of the developed SNM discrimination system to PM/MM pairs of A>T substitution at position 7 at different temperatures. The concentrations of both PM and MM were 20 nM. Each experiment was repeated three times.
